# Examining the Role of Matrix Metalloproteinase-2 and MicroRNAs Regulation in Breast Cancer

**DOI:** 10.1155/tbj/8816789

**Published:** 2025-11-29

**Authors:** Elmira Aboutalebi Vand Beilankouhi, Niloufar Kheradi, Yosra Vaez-Gharamaleki, Salomeh Roshani, Sana Abbasi, Reza Safaralizadeh, Mohammad Valilo

**Affiliations:** ^1^Department of Animal Biology, Faculty of Natural Sciences, University of Tabriz, Tabriz, East Azerbaijan, Iran; ^2^Molecular Medicine Research Center, Tabriz University of Medical Sciences, Tabriz, East Azerbaijan, Iran; ^3^Hematology and Oncology Research Center, Tabriz University of Medical Sciences, Tabriz, East Azerbaijan, Iran; ^4^Department of Biochemistry, Faculty of Medicine, Urmia University of Medical Sciences, Urmia, West Azerbaijan, Iran

**Keywords:** breast cancer, miRNA, MMP-2, molecular pathways

## Abstract

Matrix metalloproteinase (MMPs) is a class of zinc-dependent enzymes that play an important role in the invasion and metastasis of cancer cells and have different types. MMP-2 is one of the important enzymes of this family. MicroRNAs (miRNAs) are noncoding RNAs that are involved in the regulation of gene expression of many enzymes and factors in the body. Emerging data have highlighted the relationship between MMP-2 and miRNAs. Studies have shown that miRNAs regulate MMP-2 by binding to the 3′ untranslated region (3′ UTR), which leads to a decrease or increase in MMP-2 expression and its enzymatic activity. For example, decreased expression of miR-106b leads to increased growth and invasion of breast cancer (BC) cells through increased expression of MMP-2. Therefore, understanding the regulatory mechanisms related to MMP-2 and miRNAs will provide new insights into the molecular pathways that drive BC progression and highlight potential therapeutic targets for the management of invasion and metastasis. Hence, in this study, we aimed to elucidate the relationship between MMP-2 and miRNAs in BC.

## 1. Introduction

Breast cancer (BC) is a prevalent type of cancer in women, responsible for approximately 16% of cancer-related fatalities in this demographic [1, 2]. Based on its molecular profile, this cancer is categorized as Luminal A, Luminal B, basal-like, human epidermal growth factor receptor 2 (HER2)-enriched, and normal-like, with triple-negative BC (TNBC) being the most aggressive subtype [3, 4]. Risk factors for BC include older age, family history, obesity, high alcohol consumption, and genetic mutations [5–9]. Moreover, the impact of tumor stage and the expression of estrogen receptor (ER), progesterone receptor (PR), and HER2 on prognosis has led to a shift toward molecular-targeted therapies in treatment [10–12].

MicroRNAs (miRNAs) are short, single-stranded, noncoding RNAs, and 19–24 nucleotides long, that can degrade and inhibit translation of miRNA by binding to target messenger RNA (mRNA) [13, 14]. They are often aberrantly expressed in various cancers and can function as either oncogenes or tumor suppressors, depending on the genes they regulate [15, 16]. Disruption of miRNA expression influences genes related to differentiation, proliferation, apoptosis, resistance, migration, and invasion, impacting cell progression, including BC [17, 18].

Matrix metalloproteinases (MMPs) are zinc endopeptidases that degrade the extracellular matrix (ECM) and are crucial in processes like wound healing, inflammation, metastasis, and cancer invasion [19, 20]. MMP-2 and MMP-9 are particularly significant in cancer metastasis as they primarily degrade gelatin and collagen IV and V, enhancing the invasiveness of cancer cells [21, 22]. The expression of MMPs is regulated by several mechanisms including miRNAs-directed regulation [23]. MMP-2, specifically, is vital in BC cell invasion, marking it as a key biomarker for BC risk [24]. This article explores the regulatory roles of miRNAs and MMPs in the gene expression and invasion of BC cells.

## 2. BC Overview

BC is the most common form of cancer, accounting for nearly 25% of all cancer cases in women worldwide. With approximately 23 million new cases diagnosed each year, nations have yet to take significant action against this major health threat, as research has shown. This trend of increasing BC is increasing in every country, and Iran is no different, as BC is one of the most common tumors [25–28]. According to the Ministry of Health in the US, BC is the leading cancer prevalence of all the incidences that have been reported across different origins, and it is diagnosed in approximately one case out of every four new horizontally malignant neoplasms, eleven percent of which are screen positive. Diagnosis: There are five pillars or other options for BC management, and early-stage BC makes you eligible for a punch biopsy [27, 29]. Mammography: Traditionally, screening for BC has been used, which is often variant and is optimally sensitive for detecting cancers. Ultrasound: That would also explain some of the mammography depicted as well. Biopsy: In this regard, the sectioning also has a more tactical purpose because it involves detailed screening of diseased breasts and deploying intrudes for diseased areas. Magnetic resonance imaging (MRI): Magnetic images can be useful in extremely infrequent times for detailed viewing of the breast in a noninvasive way [27, 30, 31].

Treatment: The type and stage of diagnosis differ concerning actual treatment modalities for the disease, which are usually: Surgery: used to determine the BC staging and to selectively remove the cancerous mass using lumpectomy or complete mastectomy. Radiation Therapy: A method used in markets to remove any residual tumors that are left after an incision procedure. Breast carcinoma remains a common cause of cancer-related morbidity and mortality in women worldwide [13, 30].

## 3. miRNA Regulation in BC

miRNAs are small RNA molecules that do not code proteins. They play a crucial role in the post-transcriptional regulation of several genes and are essential for cellular functions such as the cell cycle, differentiation, and apoptosis [32–34]. The intergenic region is transcribed by RNA polymerase II to generate primary miRNA (pri-miRNA), typically 3000 to 5000 bases long. Most miRNA genes are transcribed in the nucleus by RNA polymerase II, resulting in capped, spliced, and polyadenylated pri-miRNAs [35, 36]. This pri-miRNA undergoes asymmetric cleavage by RNase III Drosha and its cofactor DGCR8, resulting in precursor miRNA (pre-miRNA), about 70 nucleotides long. The pre-miRNA is then transported to the cytoplasm via exportin 5 and RanGTP, where Dicer cleaves it into 22 nucleotide fragments, forming duplex molecules. One mature strand associates with AGO proteins to create the RNA-induced silencing complex (RISC) [35, 37].

A single miRNA can target multiple mRNAs, with interaction efficacy influenced by subcellular localization, mRNA abundance, and binding affinity. Numerous studies highlight the crucial role of miRNAs in human cancers, showcasing their potential as biomarkers for diagnosis, prognosis, and therapeutic applications [38, 39].

Advances in molecular biology have extensively expanded the understanding of the contribution of miRNAs to breast carcinogenesis and cancer progression. We showed that some of miRNAs are not found in the breast tissues of BC patients, one of them showing exactly the same distinct pattern, and the others absent in something completely different. For instance, miR-21 is generally highly expressed in BC patients [36, 40].

Similarly, oncomiRs such as miR-145 and miR-200c, which are normally under-expressed in BCs, induce growth and metastatic spread of cancer by estrogen. As diagnostic and prognostic markers of such neoplasms, BCs also impact their corresponding the miRNAs through dysregulation. For example, some miRNAs are present in the body fluids or tissues and are called disease markers (i.e., a marker of disease or disease stage) because the concentration of some miRNAs is increased. For example, a published study reports that circulating levels of miR-155 are increased in the case of BC, and levels are related to the site of the neoplasm in the body to allow for a noninvasive assessment of the cancer [41, 42].

miRNAs can act as either oncogenes or tumor suppressors, depending on their target genes [43, 44].

Alternations in the expression of miRNAs in various malignancies have led to their use as an important factor in diagnosing or treating cancers, especially BC, regardless of their oncogene or inhibitory role [45]. For example, elevated levels of miR-21 in BC correlate with poor prognosis, indicating that this miRNA can serve as a predictor of outcomes in BC cases [45, 46]. Altered miRNA expression significantly influences cancer progression by impacting angiogenesis, cell proliferation, apoptosis, and migration [47].  Table 1 outlines various miRNAs associated with BC and their effects.

## 4. MMPs

MMPs are a family of zinc-dependent endopeptidases, which degrade components of the ECM and are essential for tissue remodeling, wound healing, and cell migration. MMPs are involved in a variety of aspects of tumor progression in BC including invasion, metastasis, and angiogenesis. Overexpression of these proteins is associated with poor prognosis and aggressive cancer behavior [48].

MMP expression and activity in BC are regulated by a complex interplay of intrinsic and extrinsic factors:

Hormonal signaling: It has been shown that estrogen and progesterone upregulate MMP expression in BC cells. However, the transcription of MMP genes is subject to the influence of ER and PR and can promote tumor invasiveness [49].

Growth factors: Potent regulators of MMP activity include Transforming Growth Factor-beta (TGF-β), Epidermal Growth Factor (EGF), and Vascular Endothelial Growth Factor (VEGF). These growth factors activate signaling pathways to increase MMP production which promotes tumor expansion and angiogenesis [49].

Inflammatory cytokines: The inflammatory microenvironment of tumors (including cytokines such as Interleukin-1 (IL-1) and Tumor Necrosis Factor-alpha (TNF-α) has been shown to stimulate MMP secretion and increase cancer cell invasion [50].

Genetic and epigenetic factors: In addition, dysregulation of MMPs in BC can be due to genetic mutations and epigenetic modifications. The aggressive forms of BC often have altered expression of MMPs due to mutations of regulatory genes or promoter regions [51, 52].

## 5. MMPs in the Tumor Microenvironment (TME)

MMP regulation is critical to the TME. Cancer cells interact with stromal cells, including fibroblasts, immune cells, and endothelial cells, and these stromal cells produce and activate MMPs. In particular, CAFs are the major sources of MMPs in the TME. They secrete growth factors, cytokines, and proteases to promote tumor progression and metastasis. Furthermore, MMP activity may also be influenced by the TME via modulation of the balance between MMPs and their inhibitors, the tissue inhibitors of metalloproteinase (TIMPs). Uncontrolled ECM degradation and tumor progression are enabled by a reduced ratio of TIMPs to MMPs [53, 54].

## 6. MMP-2 and Its Function in BC

As mentioned above, MMPs break down ECM components, such as collagen, fibronectin, and Laminin, which act as a structural barrier and limit cancer cell migration and metastasis. MMPs degrade these ECM proteins, allowing cancer cells to break out of surrounding tissues and enter the bloodstream, spreading the tumor cells to distant organs. This process is an important step in cancer metastasis, the cause of most cancer-related deaths. Several MMPs, including MMP-2, MMP-9, and MMP-14, are particularly implicated in BC progression [55].

Degradation of type IV and type V collagen, key components of the basement membrane, requires MMP-2 (gelatinase A) and MMP-9 (gelatinase B) [56]. MMP-2 and MMP-9 are often found at elevated levels in the blood and tissues of patients with invasive BC, and those with the highest levels tend to be associated with more metastatic cancer. MMP-14 (MT1-MMP) is a membrane-bound enzyme that activates proMMP-2 for ECM degradation at the cell surface and facilitates cancer cell invasion [40].

MMP-2 is a gelatinase A, type IV collagenase, that consists of three fibronectins type II repeats that allow for degradation of denatured type IV collagen (gelatin) and elastin. Unlike most MMP family members, activation of this protein can occur on the cell membrane. This enzyme can be extracellularly activated by proteases and intracellularly by S-glutathionylation without requiring the pro-domain to be removed by proteases [57]. Studies revealed that this potential biomarker circulates in three forms: active (aMMP-2), latent (pro-MMP-2), and total (tMMP-2). The level of latent form in BC patients was reported higher in comparison to the healthy group; however, the active form was reported lower in the BC group which led to a lack of a significant association between the level of total form of BC patients and healthy patients [58].

Nodulosis-Arthropathy-Osteolysis (NAO) syndrome along with Winchester syndrome has been attributed to mutations in the MMP-2 gene. Alternative splicing leads to numerous transcript variants encoding distinct isoforms [59]. To this day several MMP-2 gene polymorphisms have been studied and related to various malignancies such as lung, cervical, prostate, and BC. However, Dofara et al. after reviewing 37 polymorphisms of MMP-2 gene and BC concluded that most of them were not associated with BC [58].

Previous reports show that MMPs have been associated with neoplastic processes for a long time, particularly in the central nervous system (CNS) [60]. The first evidence was the high expression of MMP-2 in astrocytes when they were cultured with glioma cells, and the invasive nature of glioma was attributed to it [61, 62]. The astrocytes were also implied to have a role in the brain metastasis of BC patients [63]. Tumoral MMP-2, unlike stromal MMP-2, was associated with reduced overall survival (OS) and a higher risk of distant metastasis in BC patients. Serum level of MMP-2 is also reported to be a potential biomarker for poor prognosis in pre- and postoperative BC patients [64]. There are several pathways involving MMP-2 that are studied for future therapeutics in BC, including (1) TIMP2-MMP-2/-9 pathway, (2) PI3K/AKT signaling pathway, (3) elevated STAT3 signaling, and (4) inhibiting RhoA pathway in TNBC.

## 7. BC Therapy Using MMP Inhibitors (MMPIs)

MMPs have been considered potential therapeutic targets due to the critical role of MMPs in BC progression. The purpose of MMPIs is to block the enzymatic activity of MMPs in order to prevent ECM degradation, tumor cell invasion and metastasis. Clinical trials employing broad-spectrum MMPIs have been disappointing, largely because of off-target effects and the essential role of MMPs in normal tissue remodeling [65].

However, research continues to explore more selective MMPIs that would, in theory, selectively target tumor-associated MMPs without affecting normal tissue functions. Furthermore, the use of MMPIs in combination with other therapies, such as chemotherapy, immunotherapy, and targeted therapies, may be a more effective treatment strategy [66].

BC is facilitated by MMP, as it facilitates tumor invasion, metastasis and, angiogenesis. Hormonal signaling, growth factors, cytokines, and the TME all regulate their regulation. Although MMPIs have been less successful in the clinic, they are still a promising area of research, and future strategies could be directed toward more specific and effective targeting of MMPs to improve BC treatment outcomes [67].

The advent of next-generation sequencing technologies brought with it the discovery of several miRNA variants of heterogeneous lengths and/or sequences. Initially ascribed to sequencing errors/artifacts, these isoforms, named isomiRs, are now considered noncanonical variants that originate from physiological processes affecting canonical miRNA biogenesis. To date, accurate IsomiRs abundance, biological activity, and functions are not completely understood; however, the study of isomiRs biology is an area of great interest due to their high frequency in the human miRNome, their putative functions in cooperating with the canonical miRNAs, and potential for exhibiting novel functional roles. The discovery of isomiRs highlighted the complexity of the small RNA transcriptional landscape in several diseases, including cancer. In this field, the study of isomiRs could provide further insights into miRNA biology and its implication in oncogenesis, possibly providing putative new cancer diagnostic, prognostic, and predictive biomarkers as well.

## 8. Interaction of MMP-2 and miRNA in BC

miRNAs negatively regulate their targets by inhibiting translation of mRNA, usually via complementary base pairing to the 3′-UTA. Depending on the wide range of roles in the cancers, miRNAs are categorized as oncogenic miRNAs (oncomiRs) and tumor suppressor miRNAs (TS miRNAs); however, this classification may not be accurate since in numerous cases they could suppress one specific gene but promote another oncogene, which leads to confusing results.

**Figure 1:**
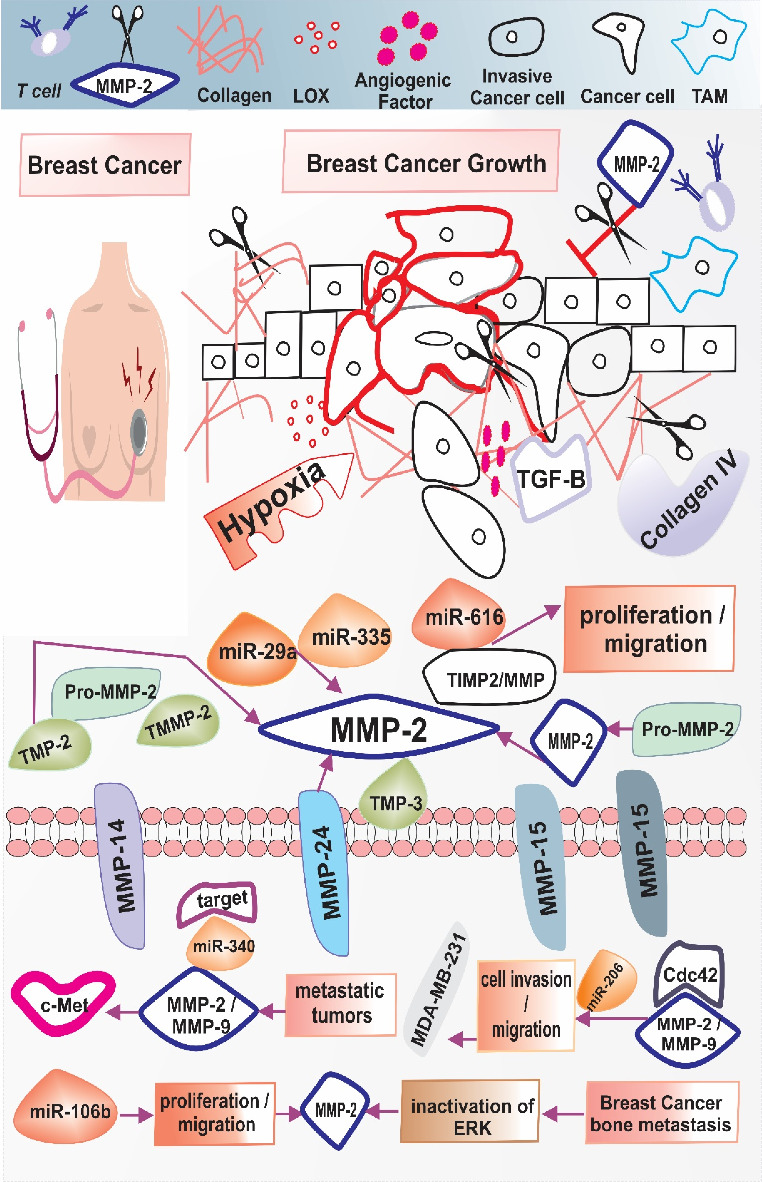
Regulatory relationships between miRNAs and MMP-2 in breast cancer progression. ECM degradation is essential for blood vessel formation and proliferation, and MMP-2 can process various ECM components, including type I collagen, to aid in these processes. The diagnostic potential of miR-29a, miR-335, and MMP-2 was evaluated, and their correlation was shown to be more effective than traditional biomarkers such as CEA and CA 15.3. Furthermore, miR-616 increased BC cell proliferation and migration by directly affecting the tissue inhibitor TIMP2, suggesting that the miR-616/TIMP2/MMP pathway could serve as a novel therapeutic target. The oncoprotein c-Met was identified as a target of miR-340 in human BC cell lines, which promotes cell migration by regulating the expression of MMP-2 and MMP-9. Consequently, decreased expression of miR-340 was associated with higher-grade cancers. In metastatic tumors, the levels of cell division control protein 42 (Cdc42), MMP-2, and MMP-9 were lower, while miR-206 may reduce the invasion and migration of MDA-MB-231 cells, in part by managing the remodeling of the actin cytoskeleton. In addition, miR-106b can inhibit cell proliferation and migration by reducing MMP-2, which inactivates ERK and increases osteoblast activity through the miR-106b/MMP-2/ERK pathway, potentially leading to bone metastasis in BC. Breast cancer: BC; TAM: tumor-associated macrophage; matrix metalloproteinase-2: MMP-2. Control protein 42: Cdc42; TIMP metallopeptidase inhibitor 2: TIMP2; extracellular signal-regulated kinase: ERK1.

Figure 1 illustrates the complex interactions between miRNAs and MMP-2 in BC progression. Recently, studies have been published that evaluated miR-29a, miR-335, and MMP-2 and examined their correlation, which was superior compared to conventional biomarkers such as CEA and CA 15.3 [68]. Yuan detected that the miR-616 increased proliferation and migration of BC cells by directly affecting the TIMP2 and emphasizing the role of miR-616/TIMP2/MMP axis as a novel therapeutic target [69]. Wu et al. detected oncoproteins c-Met as a miR-340 target in cancerous human breast cell lines, which can induce the migration of these cells through regulation of MMP-2 and MMP-9 expression, therefore, loss of miR-340 expression was associated with high-grade and metastatic tumors [70]. Liu et al. showed that levels of cell division control protein 42 (Cdc42), MMP-2, and MMP-9 were downregulated, and miR-206 may inhibit MDA-MB-231 cell invasion and migration in vitro in part via controlling actin cytoskeleton remodeling [71]. Ni et al. reported that miR-106b is capable of inhibiting proliferation and immigration by suppressing MMP-2 which leads to the inactivation of extracellular signal-regulated kinases (ERK) and increases the activity of osteoblasts via MiR106b/MMP-2/ERK pathway that can lead to BC bone metastasis [72] (Table 2).

## 9. miRNAs-Based Therapeutic Strategies in the Regulation of MMP Expression in BC

MMPs are involved in cell migration and invasion and are often regulated by various miRNAs, making them promising targets for cancer treatment. However, clinical trials of MMPIs in advanced cancer patients have consistently failed due to nonselective effects and low efficacy. Traditional methods using small-molecule inhibitors and blocking antibodies face challenges due to the structural similarities among MMP active sites and their involvement in crucial biological processes, making it difficult to develop specific inhibitors without adverse side effects [73].

MMP-2 and MMP-9 are the most extensively studied MMPs in the context of migration, invasion, and metastasis. Although more than 10 miRNAs can suppress tumor migration and invasion by regulating MMP-2 and MMP-9, only miR-29b directly targets MMP-2, and only miR-491-5p directly targets MMP-9 [73–75]. MiR-9 targets MMP-14 to inhibit neuroblastoma cell invasion, migration, and metastasis. MiR-146a/b targets MMP-16 to inhibit glioma and Caco-2 cell migration and invasion. MiR-152 targets MMP-3 to reduce glioma cell invasion, and miR-222 targets MMP-1 to decrease cell invasion in oral tongue squamous cell carcinoma (OTSCC) [75, 76].

There are two main strategies for using miRNAs in cancer therapy to regulate MMP expression: restoring tumor-suppressor miRNAs using virus-based vectors or miRNA mimics, and interfering with oncomiRs using antisense-based methods. Techniques to inhibit oncomiRs include small-molecule inhibitors of miRNAs (SMIRs), antimiRNAs (ASOs), and locked nucleic acid (LNA) antimiRs [77].

RECK, a membrane-bound glycoprotein, negatively regulates MMP-2, MMP-9, and MT1-MMP. Upregulation of miR-182 is associated with BC progression, increasing tumorigenicity and invasiveness by repressing RECK. Restoring RECK with anti-miR-182 reduces MMP-9 activity and cell invasion in BC [77, 78].

MiR-616 enhances BC cell proliferation, migration, and invasion by targeting TIMP2. The miR-616/TIMP2/MMP axis may play a role in BC progression. MiR-138 prevents BC metastasis by inhibiting the epithelial mesenchymal transition (EMT) process. Suppression of miR-138 leads to increased MMP-13 production, with a strong negative correlation between MMP-13 levels and miR-138 expression in BC. Decreased miR-138 expression also increases MMP-2 and MMP-9, promoting tumor metastasis [69].

Elmasry et al. have shown that miR-138 can prevent the metastasis of BC cells by inhibiting the EMT process. So, the suppression of miR-138 seems to lead to the production of MMP-13 in BC [79]. Elmasry et al. have shown that a strong negative correlation exists between MMP-13 levels and miR-138 expression in BC [79]. Similarly, Wang et al. [80] reported that the suppression of miR-138 leads to an increase in ECM markers like collagen I and MMP-13. In the same vein, Tan et al. [81] showed that the decreased expression of miR-138 leads to an increase in the expression of MMP-2 and MMP-9, which can degrade the ECM and promote tumor metastasis [79].

miRNAs are a promising cancer therapy option because they can regulate hundreds of genes in a tissue-specific manner and target MMPs more selectively than traditional inhibitors. Unlike traditional methods, miRNAs operate within regulatory networks, allowing simultaneous modulation of multiple molecules and more effective control of cancer-related processes [73, 75].

In summary, the number and composition of miRNAs regulating each MMP vary greatly, and the same miRNA can affect different types of MMPs, likely due to tissue-specific effects. Multiple regulatory pathways are involved in miRNA-mediated MMP regulation, many of which remain unknown. Additionally, many MMPs involved in tumorigenesis, including MMP-7, 8, 10, 12, 15, 17, 21, 22, 23, 24, 26, and 27, have not yet been shown to be regulated by miRNAs [75].

## 10. Conclusion

One of the complex regulatory mechanisms that significantly influences BC progression is the interaction between MMP-2 and miRNAs. Some miRNAs, including miR-340, can induce the migration of these cells in human BC cell lines by regulating the expression of MMP-2 and MMP-9; however, silencing of miR-340 expression is associated with high-grade and metastatic tumors. Further investigation of these interactions may uncover new strategies for treatment and highlight the importance of miRNA regulation in cancer biology. Understanding these molecular pathways offers valuable insights into the development of more effective and targeted therapies for BC patients.

## Figures and Tables

**Table 1:** The outline presents various miRNAs linked to BC and their effects.

miR	Function	Target	Effect	Ref
miR-145	Tumor suppressive	FSCN-1 MMP-2 MMP-9	- Inhibits EMT and cancer cell migration	[82]
miR-192	Tumor suppressive	RS1 EF1 PPIA	- Induces cell cycle arrest - Promotes apoptosis and DOX sensitivity	[83–85]
miR-215	Tumor suppressive	RAD54B SOX9	- Activates apoptosis and inhibits cancer cell proliferation and migration - Inhibits invasive ability of cancer cells	[86, 87]
miR-16–5p	Tumor suppressive	EPHA1	- Inhibits cell progression and EMT via inhibiting NF-κB signaling	[88]
miR-1246	Oncogenic	Cyclin G2 (CCNG2)	- Promotes migration, invasion and proliferation	[89]
miR-194	Oncogenic	Fbxw-7 SOX17	- Promotes cancer cell proliferation by upregulating cyclin E and cyclin D - Activates wnt/β catenin signaling pathway and promotes migration and invasion	[90, 91]

**Table 2:** Functional and pathological effects of the miRNA-mediated regulation of MMP-2.

MMPs	Group	MicroRNA	Investigated of microRNA on tumor development	Ref.
MMP-2/MMP-9	Gelatinases	miR-125b, miR-21, miR-143, miR-451, miR-7miR-206, miR-340, miR-9, miR-196b	Invasion and migration; proliferation and apoptosis	[75]
MMP-2	Gelatinases	miR-21, miR 39, miR-101, miR-146a, miR-26a	Invasion and migration; angiogensis	[75]
MMP-2	—	miR-106b	Migration and invasion	[92]
MMP-2 and MMP-9	—	miR-206	Invasion and migration	[71]
MMP-2 and MMP-9	—	miR-340	Tumor cell growth, migration, and invasion	[70]
MMP-2/MMP-9	Gelatinases	Upregulated (miR-155, miR-10, miR-21, and miR-373) Downregulated (miR-340 and miR-206)	Invasion and migration	[77]
MMP-2	Gelatinases	Downregulated (miR-106b)	Invasion and migration	[77]

## Data Availability

Data sharing does not apply to this article, as no new data were created or analyzed in this study.
